# Quantum Calculations of VX Ammonolysis and Hydrolysis Pathways via Hydrated Lithium Nitride

**DOI:** 10.3390/ijms22168653

**Published:** 2021-08-11

**Authors:** Calen J. Leverant, Chad W. Priest, Jeffery A. Greathouse, Mark K. Kinnan, Susan B. Rempe

**Affiliations:** 1 Sandia National Laboratories, Albuquerque, NM 87185, USA; clevera@sandia.gov (C.J.L.); chadpriest01@gmail.com (C.W.P.); jagreat@sandia.gov (J.A.G.); mkkinna@sandia.gov (M.K.K.); 2 Department of Chemical Engineering, University of Florida, Gainesville, FL 32611, USA

**Keywords:** VX, aminolysis, base hydrolysis, decontamination, Hartree–Fock, density functional theory, chemical warfare agent

## Abstract

Recently, lithium nitride (Li_3_N) has been proposed as a chemical warfare agent (CWA) neutralization reagent for its ability to produce nucleophilic ammonia molecules and hydroxide ions in aqueous solution. Quantum chemical calculations can provide insight into the Li_3_N neutralization process that has been studied experimentally. Here, we calculate reaction-free energies associated with the Li_3_N-based neutralization of the CWA VX using quantum chemical density functional theory and ab initio methods. We find that alkaline hydrolysis is more favorable to either ammonolysis or neutral hydrolysis for initial P-S and P-O bond cleavages. Reaction-free energies of subsequent reactions are calculated to determine the full reaction pathway. Notably, products predicted from favorable reactions have been identified in previous experiments.

## 1. Introduction

Organophosphates (OPs), a class of nerve agents and pesticides, are among the most toxic of chemical warfare agents (CWAs) [1]. Upon exposure, OPs attack the central nervous system, causing involuntary bodily functions such as seizures. OPs have binding affinities similar to that of the neurotransmitter acetylcholine, allowing OPs to inhibit the action of acetylcholinesterase in neuromuscular junctions, disrupting the breakdown of the neurotransmitter [2,3,4]. The neutralization of OP compounds can be achieved through oxidation [5,6], enzyme degradation [7,8,9], hydrolysis [10,11,12], and incineration [13]. Nevertheless, each method is not without its limitations. Oxidation requires large excesses of oxidizing agents and is not very selective [6,14]. Enzymes used in CWA degradation are not stable and cannot be stored for long periods of time [15]. Hydrolysis often uses a large quantity of water [14]. Finally, incineration requires that the CWAs are moved to facilities with the appropriate equipment [16], and thus cannot be used in many locations.

Of the OP CWAs, ethyl({2-[bis(propan-2-yl)amino]ethyl}sulfanyl)(methyl)phosphin-ate, or VX, is among the most toxic. Like most OPs, it contains a pentavalent central phosphorous atom, P(V). It has a four-coordinate chiral center at the P(V)-atom, with two enantiomer forms (S)-VX and (R)-VX. The structure contains a phosphoester (P-OR), a methyl (P-CH_3_), a terminal oxide (P=O), and a phosphothiol (P-SR′) bond. Here, R is an ethyl group and R′ is a complex constituent containing a (diisopropylamino)ethyl chain.

**Scheme 1:**
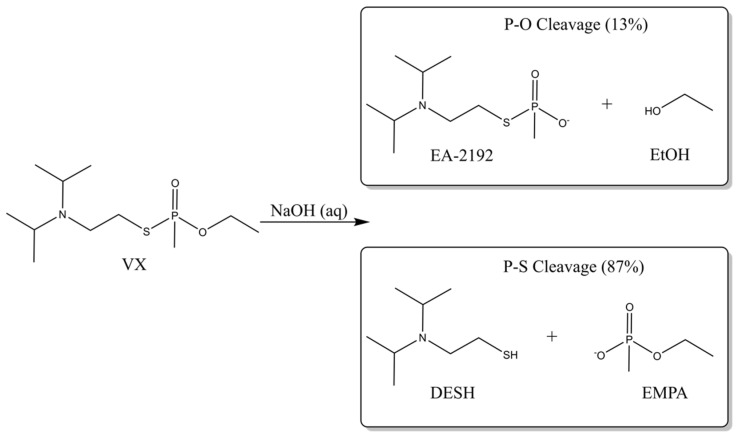
Alkaline hydrolysis of VX resulting in EA-2192 from P-O cleavage or EMPA from P-S cleavage.

Molecular modeling is a useful tool to study CWA properties such as liquid structure [17,18], adsorption [19,20,21], and reactivity [22]. Quantum methods, specifically density functional theory (DFT) and ab initio techniques, are ideally suited to investigate fundamental reaction pathways of these compounds. Understanding the mechanistic steps involved in the decomposition of toxic chemicals such as CWAs continues to be an active area of research [23]. Many computational efforts have studied decontamination of VX, or its analogues (e.g., O,S-dimethyl methylphosphonothiolate) by different chemical processes. These include neutral hydrolysis, alkaline hydrolysis, oxidation, and aminolysis reactions [10,12,22,24,25]. Most of these chemical decontamination processes rely on a nucleophile. For these compounds, nucleophilic reactions can undergo two competing solvolysis reaction pathways: P-S or P-O bond cleavage [6,26,27], shown in Scheme 1 for alkaline hydrolysis. P-O cleavage produces toxic EA-2192 (S-[2-(diisopropylamino)ethyl] hydrogen methylphosphonothioate), while P-S cleavage yields the non-toxic EMPA (ethyl methylphosphonic acid). Alkaline hydrolysis of VX yields 13% EA-2192 and 87% EMPA [6].

In earlier computational work, Patterson and coworkers used a combination of DFT and Hartree–Fock (HF) methods to study the alkaline hydrolysis of sarin and OP compounds [10,11,12]. For VX, their results showed that the reaction pathway involving P-S bond cleavage is thermodynamically favorable compared with P-O cleavage [12]. The decomposition of VX catalyzed by the hydroxide or hydroperoxide anions also showed that the P-S cleavage is the most favorable thermodynamic pathway [11]. As validation of their methods, the calculated enthalpy of activation for sarin agrees well with the experiment [12]. Mandal et al. used DFT methods to examine the P-O and P-S bond cleavage reaction pathways in the VX analogue, O,S-dimethyl methylphosphonothiolate, in the gas phase and in the presence of various implicit solvents [24]. Although previous calculations suggested that P-S bond cleavage is approximately 5–10 kcal/mol more favorable than P-O bond cleavage in similar compounds [11,12], results from Mandal et al. indicate that breaking either bond is equally favorable [24]. They also showed that within the COSMO implicit solvent model, the type of solvent had little effect on the barrier heights or net reaction energies [24].

**Scheme 2:**
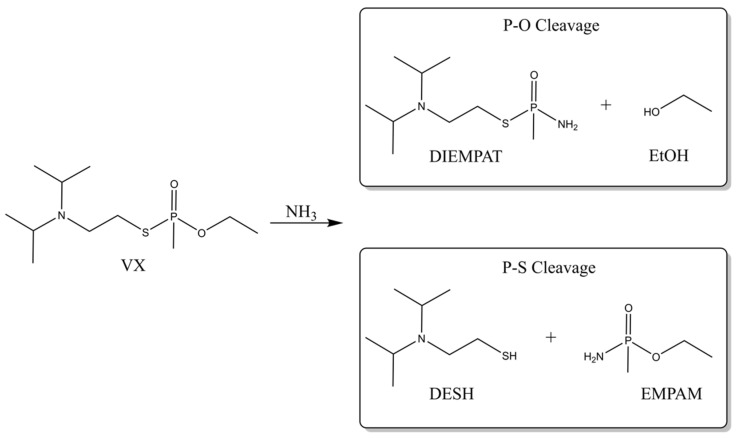
Ammonolysis of VX with NH_3_, resulting in DIEMPAT from P-O cleavage or EMPAM from P-S cleavage.

Recently, Li_3_N has been proposed as a multifunctional reagent for broadband CWA neutralization, with the objective to use a minimal amount of the reagent to disable bulk CWAs through neutralization or solidification [28,29]. When Li_3_N is hydrated, hydroxide ions and ammonia are generated, both of which can serve as nucleophiles in the decontamination of CWAs (Scheme 1 and Scheme 2). Here, hypothesized NH_3_ ammonolysis products include DIEMPAT (S-(2-(diisopropylamino) ethyl) P-methyl phosphonamidothioate) from P-O cleavage, and EMPAM (ethyl P-methyl phosphonamidite) from P-S cleavage. This work aims to explore the complete decontamination of VX from base hydrolysis and ammonolysis processes in the Li_3_N aqueous environment. These reactions can contribute toward the development of new decontamination methods and to further understand the Li_3_N CWA decontamination process.

## 2. Results and Discussions

**Figure 1:**
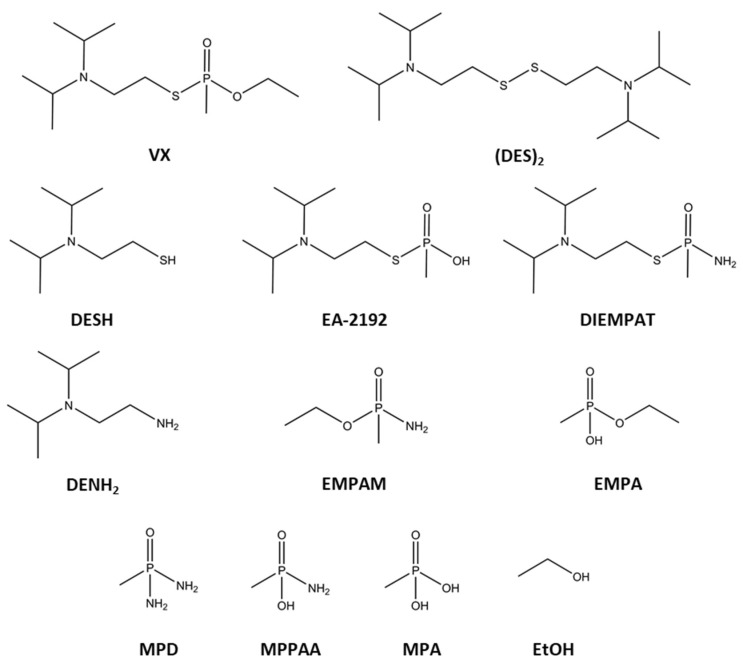
Chemical structure of VX, VX hydrolysis products, and VX ammonolysis products. VX = Ethyl ({2-[bis(propan-2-yl)amino]ethyl}sulfanyl)(methyl)phosphinate, (DES)_2_ = VX Disulfide, DESH = Diisopropylamino ethanethiol, EA-2192 = S-(2-(diisopropylamino)ethyl) O-hydrogen methylphosphonothioate, DIEMPAT = S-(2-(diisopropylamino)ethyl) P-methyl phosphonamidothioate, DENH_2_ = N,N-Diisopropylethylenediamine, EMPAM = Ethyl P-methyl phosphonamidite, EMPA = Ethyl methylphosphonic acid, MPD = P-Methylphosphonic diamide, MPPAA = P-methylphosphonamidic acid, MPA = Methylphosphonic acid, EtOH = Ethanol.

The chemical structures and the geometry-optimized structures for the VX reaction products from P-O and P-S bond cleavage are shown in Figure 1 and Figure S1 (see Supplementary Materials), respectively. All reaction pathway figures list the solvation model-based density (SMD) HF ⊗*G* values at 300 K with water as a solvent (HF-S). The gas phase HF ⊗*G* values (HF) as well as the gas phase and implicit (water) solvent B3LYP values (B3LYP and B3LYP-S) can be found in the corresponding tables.

### 2.1. Neutral Hydrolysis

**Figure 2:**
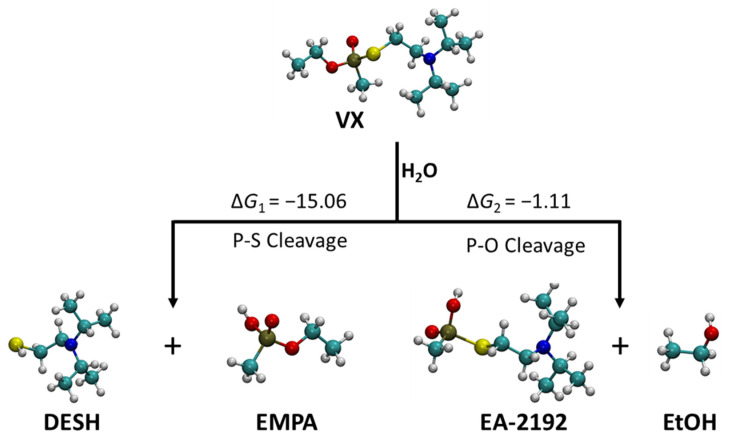
VX neutral hydrolysis pathway via P-S and P-O bond cleavages.

Small-scale experiments have shown that VX can be completely hydrolyzed into less toxic products in 30–60 days at room temperature [30]. While alkaline hydrolysis cleaves both the P-S and P-O bonds, creating a mix of toxic and non-toxic products, hydrolysis at neutral pH cleaves only the P-S bond, resulting in non-toxic products [30,31]. The neutral hydrolysis reaction energies for the VX P-S and P-O bond cleavages are shown in Figure 2. In agreement with the earlier experimental studies [30,31], we observe a much more favorable ⊗*G* value for the P-S bond cleavage as opposed to the P-O bond cleavage (−15.06 kcal/mol vs. −1.11 kcal/mol). No experimental observations have shown cleavage of P-C bonds. As shown in Table 1, similar ⊗*G* values are obtained from HF and B3LYP methods for P-O bond cleavage (⊗*G*_2_). The methods differ in P-S cleavage energy (⊗*G*_1_), with HF predicting more favorable reactions in gas and solvated phases.

### 2.2. Alkaline Hydrolysis

**Figure 3:**
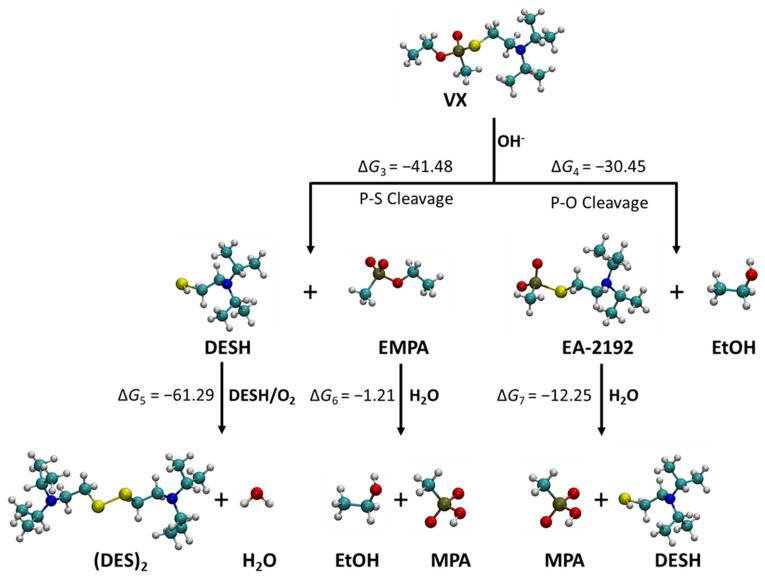
Full aqueous neutralization pathway for VX starting with an initial alkaline hydrolysis step.

Hydrolysis of VX in highly basic conditions is known to produce 87% non-toxic EMPA and 13% toxic EA-2192 (Scheme 1) [6,10]. Reaction-free energies for the initial P-O and P-S cleavage steps (Figure 3) are significantly more favorable at high pH than in neutral solution. We show the full reaction pathway for VX with OH^−^ in Figure 3. Both P-S and P-O bond cleavages are favorable, with the P-S pathway being ~11 kcal/mol more favorable. Our computed ⊗*G* values are similar to previously reported values by Daniel et al. [10].

While previous studies have looked at energy barriers and transition states of the *initial* P-S and P-O bond cleavages [10,12,24], it is important to examine the *full* reaction pathway to determine the fate of EA-2192. After the initial cleavage, EMPA retains a P-O bond and EA-2192 retains a P-S bond that may be susceptible to neutral hydrolysis, alkaline hydrolysis, or ammonolysis. While all three reaction mechanisms for EMPA and EA-2192 were considered (Table S1), only neutral hydrolysis is shown in Figure 3 for conciseness. This condition represents the scenario where most of the hydroxide ions are used up in the initial VX P-O/P-S bond cleavage, but excess water remains.

Hydrolysis of the P-S bond in EA-2192 is shown to be thermodynamically favorable, with a ⊗*G* value of −12.25 kcal/mol. This suggests that over long time periods, VX can be completely converted into non-toxic products in aqueous conditions. The organophosphorus product resulting from the P-S bond cleavage, EMPA, has a hydrolysis ⊗*G* value near zero, suggesting that it will likely remain unreacted. The additional product resulting from the P-S bond cleavage, DESH, can dimerize in the presence of oxygen to form (DES)_2_ and additional water that can participate in the hydrolysis of VX and EA-2192. However, we note that this study does not examine transition state barriers, which could play an important role in the reaction of O_2_ with DESH to form the disulfide (DES)_2_. Nevertheless, this dimerized product has been detected experimentally [28].

Table 2 shows the comparison of the B3LYP and HF methods for reactions shown in Figure 3. Both methods suggest that the P-S (⊗*G*_3_) and P-O (⊗*G*_4_) bond cleavages are thermodynamically favorable. While the magnitude of the difference in ⊗*G* value of the initial P-S and P-O bond cleavages is large, both methods suggest that the P-S bond cleavage is ~10 kcal/mol more favorable than the P-O bond cleavage. As a result, both methods are consistent with the experimental studies that report an 8:1 ratio of P-S to P-O bond cleavage during alkaline hydrolysis [6,28]. For the hydrolysis of EA-2192 and EMPA, both methods give similar ⊗*G* values. As noted previously, the B3LYP/MIDI method is known to produce accurate geometries, but not necessarily accurate reaction energies. Previously, benchmarking showed that MIDI did in fact provide accurate geometries, but needed to be replaced with MP2/6–31+G(d) for accurate energy calculations [12]. Therefore, we expect that HF provides the more reliable energy calculations for this system. Although the two methods yield significantly different ⊗*G* values in some cases, they both yield the same favorable reaction pathways.

### 2.3. Ammonolysis

**Figure 4:**
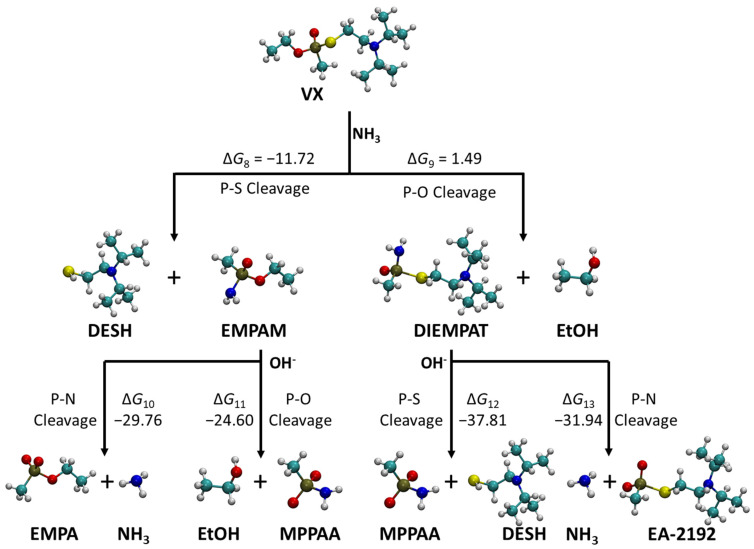
Full aqueous neutralization pathway for VX starting with an initial ammonolysis step.

While multiple previous studies have looked at the alkaline hydrolysis of the P-S and P-O bonds in VX, studies of the ammonolysis of VX are less common and have used related compounds rather than VX itself [24]. Ammonolysis of the P-S and P-O bonds of VX produce EMPAM and DIEMPAT, respectively. Out of all three reaction mechanisms, hydrolysis, alkaline hydrolysis, and ammonolysis, the latter has the least favorable ⊗*G* values for cleaving the P-S and P-O bonds (Figure 4). As shown in Table 3, both HF and B3LYP methods show that ammonolysis of the P-O bond is unfavorable (⊗*G*_9_); however, the methods conflict on the P-S bond cleavage (⊗*G*_8_). Here, HF results in a modestly favorable ⊗*G* of −11.72 kcal/mol while B3LYP results in an unfavorable ⊗*G* of 3.88 kcal/mol. Despite the favorable reaction predicted by HF, ammonolysis of the P-S bond is unlikely to have a significant effect on the overall neutralization of VX due to ammonolysis being the least-favorable reaction pathway.

Although the reaction of VX with NH_3_ was calculated to be either unfavorable (B3LYP) or only slightly favorable (HF), subsequent reactions were studied for completeness and for comparison with NMR and LC/MS product identification experiments. Once again, the products of the initial VX reaction can undergo neutral hydrolysis, alkaline hydrolysis, or ammonolysis; however, Figure 4 only shows alkaline hydrolysis for conciseness. Ammonolysis reaction-free energies of EMPAM and DIEMPAT are shown in Table S2. There are two possible routes each for alkaline hydrolysis of EMPAM and DIEMPAT. For EMPAM, the first is P-O bond cleavage, creating MPPAA and ethanol (EtOH). The second alkaline hydrolysis route involves attack of the P-N bond, resulting in EMPA and regenerating NH_3_. For DIEMPAT, alkaline hydrolysis can occur at the P-S bond, producing MPPAA and DESH, or at the P-N bond, creating EA-2192 and regenerating NH_3_. Both HF and B3LYP methods predict that all four routes of alkaline hydrolysis of EMPAM and DIEMPAT are favorable. Similar to EMPA and EA-2192 reactions (Table 2), the magnitude of the ⊗*G* value predicted by B3LYP is much larger than that predicted by HF. It is also important to note that ammonolysis of VX followed by alkaline hydrolysis of the P-N bond produces the same products as alkaline hydrolysis of VX initially. If alkaline hydrolysis of the P-N bond is significantly faster than ammonolysis, it could be difficult to verify this ammonolysis pathway experimentally with a complex reaction mixture.

### 2.4. Computational and Experimental Agreement

**Figure 5:**
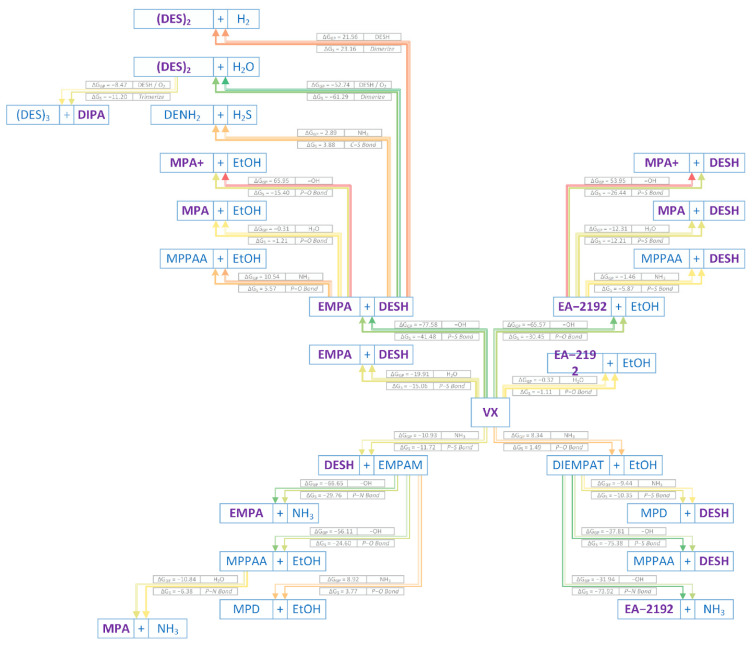
Reaction pathways for the complete degradation of VX in an alkaline environment. Reaction-free energies were obtained from HF calculations in gas phase (GP) and solvent (water, S) environments. Compounds in purple indicate species that have been detected experimentally. Reaction arrows are colored according to the reaction free energy values (green: Favorable, yellow: Slightly favorable or unfavorable, red: Unfavorable).

Figure 5 shows expected products and reaction free energies for the degradation of VX in alkaline conditions. Results are shown for calculations in the gas phase and with implicit solvent (water). Products shown in purple have been verified experimentally using the techniques P-31 NMR, C-13 NMR, and LC/MS [28]. In this reaction, 8 g of Li_3_N tablets and 10 mL of water were added to 100 mL of VX. The VX was reduced to 6.6 wt% after one week and was undetectable after 3 months [28]. Both NMR techniques and LC/MS results confirmed the presence of EMPA. EA-2192 was identified by P-31 NMR and LC/MS, VX Disulfide by C-13 NMR, and diisoproylamine (DIPA) by C-13 NMR and LC/MS. Nevertheless, the presence of an experimentally verified product does not always ensure that a specific reaction has occurred as many routes can lead to the same products.

Cleavage of either the P-S or P-O bond by OH^−^ is predicted to be energetically favorable, and confirmed experimentally by the presence of intermediates EMPA, DESH, and EA-2192 in the reaction mixture. The subsequent reactions of EA-2192 and EMPA with OH^−^ are generally less favorable and may occur at a slower rate. The dimerization of DESH to form the (DES)_2_ is predicted to be favorable. The observation of (DES)_2_ and DIPA substantiates the computational observation that oligomers do form as the reaction progresses. No additional computational reactions were performed to investigate continued oligomerization beyond three monomer units. The reaction of VX with NH_3_ is either unfavorable or only slightly favorable, as discussed above. However, alternative ammonolysis pathways resulting in NH_3_ could result in an autocatalytic process but a mechanism to increase the favorability of these reactions needs to be developed. Additional calculations to identify transition states for promising reaction pathways could be performed to obtain kinetic information for the initial steps. However, such calculations are beyond the scope of this work, whose purpose is to screen a large number of potential reaction pathways.

## 3. Materials and Methods

The reaction pathways were investigated with quantum mechanical calculations using the Gaussian 16 software suite [32]. The pathways examined include VX reactions with the nucleophilic hydroxide ions and ammonia molecules generated during the hydration of Li_3_N, rather than explicitly modeling Li_3_N itself. All optimized geometries and harmonic frequencies were calculated using the HF level of theory for consistency with previous calculations on hydrolysis of similar compounds [10,11,12]. HF methods have been shown to predict reaction barrier heights poorly [33]. However, in this work, we use HF to compare reaction thermodynamics for stable intermediates and end products for several solvolysis mechanisms. We use an augmented polarized double zeta basis set (6–31 + G(d,p)) [10], which is important to describe chemically inequivalent orbitals accurately. This basis set is considered one of the best double-zeta basis sets available [34]. For comparison, calculations were also performed at the DFT level of theory using the B3LYP functional with the MIDI! basis set, a computationally cheaper combination of valence basis functions and polarization functions that gives geometries and charges comparable to Moller–Plesset methods [35]. The B3LYP calculations included the empirical dispersion GD3BJ dampening function to account for nonbonded interactions [36]. Thermodynamic data were obtained from frequency calculations. The vibrational modes were checked for imaginary frequencies to ensure the optimized geometries were at local minima. Reaction-free energies, ⊗*G*, were calculated from the difference in free energies between the sum of reactants and products. To account for solvent effects and to more closely match experimental reaction studies, subsequent self-consistent reaction field (SCRF) analysis from the SMD implicit approximation with dielectric of water (ε = 78.35) was used for the HF calculations [37]. For the DFT calculations, the polarizable continuum model (PCM) was used to account for the solvent effects of water. In the solvated ammonolysis reactions, the free energy of ammonia is calculated in the solvated state, not the gas-phase. Therefore, no change in standard state from gas-phase to solution phase occurs. Molecular models were created using VMD [38].

## 4. Conclusions

The combination of Li_3_N and water appears to be a promising method for the neutralization of bulk CWA fluids [28,29]. To provide molecular-level insight into potential reactions involved in this process, we studied the neutralization of CWA VX by neutral hydrolysis, alkaline hydrolysis, and ammonolysis using ab initio and DFT methods. Our calculations show alkaline hydrolysis as the most favorable mechanism, with a ~10 kcal/mol preference for the P-S over the P-O cleavage for all methods. In aqueous solutions at the HF level of theory, we observe a slightly favorable ⊗*G* for the P-S bond, but not the P-O bond, for both ammonolysis and neutral hydrolysis. This suggests that, over long periods of time, either water or NH_3_ can react with VX to produce exclusively non-toxic products. Finally, we studied further reactivity of the VX reaction products to determine the full reaction pathway. Those results were found to be in good agreement with, and complementary to, experimental studies. Many experimental products can be formed by multiple different reaction pathways, making it difficult to verify the reaction pathway from experiments alone.

## Figures and Tables

**Table 1:** Reaction-free energies (kcal/mol) for various computational methods for the reaction pathways 1 and 2 in Figure 2.

	HF-S ^a^	HF	B3LYP-S ^a^	B3LYP
⊗*G*_1_	−15.06	−19.91	−12.27	−12.54
⊗*G*_2_	−1.11	−0.32	−1.08	0.33

^a^ Calculations with implicit solvent background.

**Table 2:** Reaction-free energy comparison for various computational methods for the reaction pathways 3–7 in Figure 3.

	HF-S	HF	B3LYP-S	B3LYP
⊗*G*_3_	−41.48	−77.58	−87.18	−114.18
⊗*G*_4_	−30.45	−65.58	−78.87	−112.15
⊗*G*_5_	−61.29	−52.74	−42.84	−41.28
⊗*G*_6_	−1.21	−0.31	−0.39	2.08
⊗*G*_7_	−12.25	−12.31	−8.71	0.05

**Table 3:** Reaction free energy comparison for various computational methods for the reaction pathways 8–13 in Figure 4.

	HF-S	HF	B3LYP-S	B3LYP
⊗*G*_8_	−11.72	−10.93	3.88	5.05
⊗*G*_9_	1.49	8.34	14.32	16.61
⊗*G*_10_	−29.76	−66.65	−91.06	−119.22
⊗*G*_11_	−24.60	−56.11	−73.08	−98.64
⊗*G*_12_	−37.81	−75.38	−93.19	−128.76
⊗*G*_13_	−31.94	−73.92	−83.52	−110.20

## Data Availability

The data presented in this study are available on request from the corresponding author.

## Supplementary Materials

The following are available online at https://www.mdpi.com/article/10.3390/ijms22168653/s1.
