# Supplementary material for: Quantum Calculations of VX Ammonolysis and Hydrolysis Pathways via Hydrated Lithium Nitride
Source: Int J Mol Sci. 2021 Aug 11;22(16):8653. doi: 10.3390/ijms22168653 (PMC8395404; doi:10.3390/ijms22168653)
Supplement: Supplementary file 1 [file ijms-22-08653-s001.zip › ijms-1280062-supplementary.pdf]

Supplementary Information: Quantum Calculations of VX Ammonolysis and Hydrolysis Pathways via Hydrated Lithium Nitride

Calen J. Leverant [1,2], Chad W. Priest [1], Jeffery A. Greathouse [1], Mark K. Kinnan [1], and Susan B. Rempe [1,*]

1 Sandia National Laboratories, Albuquerque, New Mexico 87185, United States
2 Department of Chemical Engineering, University of Florida, Gainesville, Florida 32611, United States
* Correspondence: slrempe@sandia.gov

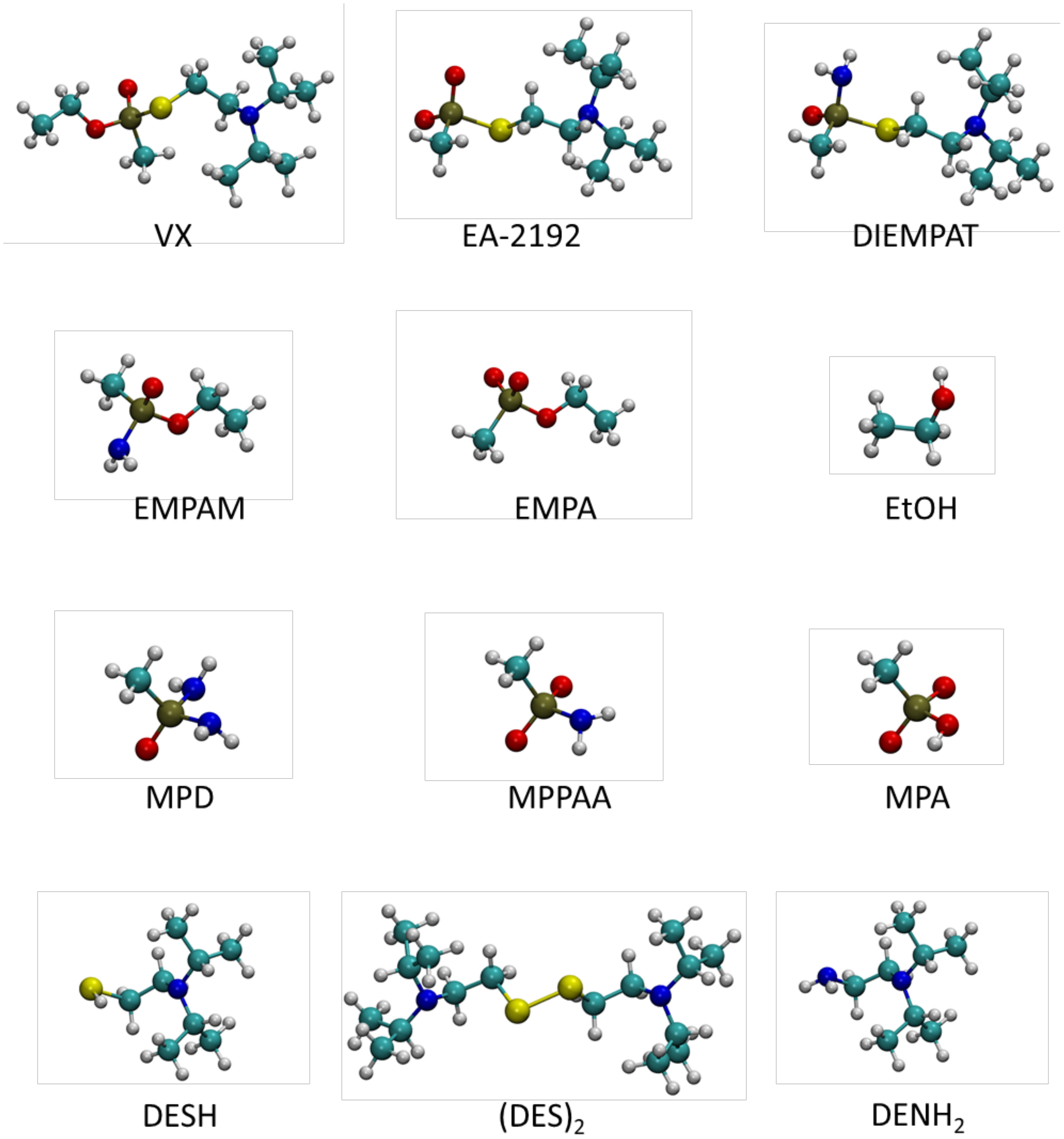

Figure S1. Optimized structure of VX, VX hydrolysis products, and VX ammonolysis products. Atoms are colored C (turquoise), H (white), O (red), N (blue), P (gray), and S (yellow).

Table S1. Reaction energy comparison for various computational methods for the reaction of EMPA and EA-2192 with H_2O ($\Delta G_{S1}/\Delta G_{S4}$), OH^- ($\Delta G_{S2}/\Delta G_{S5}$), and NH_3 ($\Delta G_{S3}/\Delta G_{S6}$)*

	Reactant	Nucleophile	HF-S	HF	B3LYP-S	B3LYP
ΔG_{S1}	EMPA	H_2O	-1.21	-0.31	-0.39	2.08
ΔG_{S2}	EMPA	OH^-	-15.40	65.95	-41.82	43.85
ΔG_{S3}	EMPA	NH_3	5.16	10.54	17.98	20.59
ΔG_{S4}	EA-2192	H_2O	-12.25	-12.31	-8.71	0.05
ΔG_{S5}	EA-2192	OH^-	-26.44	53.95	-50.13	41.83
ΔG_{S6}	EA-2192	NH_3	-5.87	-1.46	9.67	18.57

*EMPA and EA-2192 can undergo neutral hydrolysis ($\Delta G_{S1}/\Delta G_{S4}$), alkaline hydrolysis ($\Delta G_{S2}/\Delta G_{S5}$), and ammonolysis ($\Delta G_{S3}/\Delta G_{S6}$) in the aqueous Li_3N environment.

Table S2. Reaction energy comparison for various computational methods for the reaction of EMPAM and DIEMPAT OH^- via P-N cleavage ($\Delta G_{S7}/\Delta G_{S10}$), OH^- via P-O/P-S cleavage ($\Delta G_{S8}/\Delta G_{S11}$), and NH_3 ($\Delta G_{S9}/\Delta G_{S12}$)*

	Reactant	Nucleophile	Target Bond	HF-S	HF	B3LYP-S	B3LYP
ΔG_{S7}	EMPAM	OH^-	P-N	-29.76	-66.65	-91.06	-119.22
ΔG_{S8}	EMPAM	OH^-	P-O	-24.60	-56.11	-73.08	-98.64
ΔG_{S9}	EMPAM	NH_3	P-O	3.77	8.92	14.14	15.56
ΔG_{S10}	DIEMPAT	OH^-	P-N	-31.94	-73.92	-93.19	-128.76
ΔG_{S11}	DIEMPAT	OH^-	P-S	-37.81	-75.38	-83.52	-110.20
ΔG_{S12}	DIEMPAT	NH_3	P-S	-9.44	-10.25	3.71	4.01

*EMPAM and DIEMPAT can undergo alkaline hydrolysis of the P-N bond ($\Delta G_{S7}/\Delta G_{S10}$), alkaline hydrolysis of the P-O/P-S bond ($\Delta G_{S8}/\Delta G_{S11}$), and ammonolysis of the P-O/P-S bond ($\Delta G_{S9}/\Delta G_{S12}$) in the aqueous Li_3N environment.
